# IMRT treatment plans and functional planning with functional lung imaging from 4D-CT for thoracic cancer patients

**DOI:** 10.1186/1748-717X-8-3

**Published:** 2013-01-02

**Authors:** Tzung-Chi Huang, Chien-Yi Hsiao, Chun-Ru Chien, Ji-An Liang, Tzu-Ching Shih, Geoffrey G Zhang

**Affiliations:** 1 Department of Biomedical Imaging and Radiological Science, China Medical University, 91, Hsueh-Shih Road, Taichung City, Taiwan; 2 Division of Radiation Therapy & Oncology, Shin Kong Wu Ho-Su Memorial Hospital, Taipei City, Taiwan; 3 Radiation Oncology, China Medical University Hospital, Taichung City, Taiwan; 4 Radiation Oncology, Moffitt Cancer Center, Tampa, FL, USA

**Keywords:** 4D-CT, Deformable image registration, Functional lung, IMRT treatment plan

## Abstract

**Background and purpose:**

Currently, the inhomogeneity of the pulmonary function is not considered when treatment plans are generated in thoracic cancer radiotherapy. This study evaluates the dose of treatment plans on highly-functional volumes and performs functional treatment planning by incorporation of ventilation data from 4D-CT.

**Materials and methods:**

Eleven patients were included in this retrospective study. Ventilation was calculated using 4D-CT. Two treatment plans were generated for each case, the first one without the incorporation of the ventilation and the second with it. The dose of the first plans was overlapped with the ventilation and analyzed. Highly-functional regions were avoided in the second treatment plans.

**Results:**

For small targets in the first plans (PTV < 400 cc, 6 cases), all V5, V20 and the mean lung dose values for the highly-functional regions were lower than that of the total lung. For large targets, two out of five cases had higher V5 and V20 values for the highly-functional regions. All the second plans were within constraints.

**Conclusion:**

Radiation treatments affect functional lung more seriously in large tumor cases. With compromise of dose to other critical organs, functional treatment planning to reduce dose in highly-functional lung volumes can be achieved

## Introduction

Radiotherapy that avoids or reduces radiation dose in highly-functional lung regions might allow pulmonary toxicity reduction to improve survival of lung cancer patients [1]. Preservation of functional lung is becoming a focus in radiation oncology now. Radiation treatment planning with the incorporation of functional lung images to decrease radiation dose in highly-functional lung regions was reported in previous studies [2-8]. The feasibility of using lung perfusion information obtained from single photon emission computed tomography (SPECT) has been investigated previously [4,5]. The identification of good functional lung ventilation using SPECT coupled with intensity modulated radiation therapy (IMRT) is an attractive strategy [6]. The incorporation of simulation from SPECT or 3He-MRI into IMRT planning for non-small cell lung cancer (NSCLC) was studied [2]. The incorporation of four-dimensional computed tomography (4D-CT) ventilation information is the latest technology reported in several studies for radiotherapy planning purposes [3,7,8]. These studies focused on NSCLC of small targets in which a flexible beam arrangement can be easily made in functional treatment planning. The functional planning with IMRT for large thoracic tumors, including lung and esophagus cancers, are absent in studies so far. The retrospective research on dose distribution in the highly-functional lung regions was not performed.

Different imaging modalities are currently used clinically for pulmonary ventilation evaluation. Nuclear medicine, including nuclear scintigraphy [9], SPECT [10], positron emission tomography (PET) [11], is the most commonly used modality. Magnetic resonance imaging (MRI) [12] and CT [13] are also capable of pulmonary functional imaging. However, these approaches are not practically applied in radiotherapy because the additional imaging settings would increase treatment cost and patient waiting time. Lately, ventilation imaging calculated using deformable image registration (DIR) with 4D-CT becomes a hot topic [14-16]. Lung scans of the whole respiration cycle are available by 4D-CT which provides the structural tissue locations in different time-phases. With the aids of DIR, linking phase to phase of CT sets for motion estimation of lung tissues in the respiration cycle, ventilation information can be acquired. Castillo *et al.* generated ventilation image using the Hounsfield unit change or Jacobian determinant of the deformation field to estimate the local volume changes. The measurement of regional ventilation change in lung due to radiotherapy using 4D-CT and DIR techniques was reported by Ding *et al.*[17]. The 4D-CT ventilation image provides 3D high resolution ventilation information which can by incorporated in treatment planning to maximize the preservation of functional lung and improve the radiation treatment for thoracic cancer patients.

The primary purpose of this study was to evaluate the radiation dose of IMRT treatment plans on functional lung volumes and to generate IMRT plans by the incorporation of functional lung imaging from 4D-CT for thoracic cancer patients. With functional lung regions avoided by beam angle optimization and IMRT inverse-planning, thereby presumably minimizing the risk of both acute and late complications is achieved. Various tumor sizes were included in this study. The dosimetric parameters in the treatment plans were quantitatively compared.

## Material and methods

### Patient selection

Eleven patients who had non-trivial but stable respiration motion and received 4D-CT (PET/CT-16 slice, Discovery STE, GE Medical System, Milwaukee, Wisconsin USA) imaging were included in this retrospective study. The clinical characteristics of these eleven patients were presented in Table 1. Five NSCLS, one small cell lung cancer (SCLC), three esophagus cancer and two thymoma cancer patients of 8 males and 3 females made up the patient study group. The mean age of the study group was 58 (range from 49 to 72). The range of planned target volume (PTV) was from 62 to 805 cm^3^. The study group was divided to two subgroups by PTV size. The subgroup with the small PTV volume (≤400 cm^3^) consisted of 6 individuals. The mean volume was 217.6 cm^3^ ranging from 62.1 to 393.2 cm^3^. The subgroup with PTV volume exceeding 400 cm^3^ included 5 subjects with a mean volume of 710.9 cm^3^, ranging from 639.8 to 804.9 cm^3^.

**Table 1:** Characteristics of study subjects and lesions

**Patient**	**Age(y)**	**Histologic type**	**Stage**	**Location**	**PTV(cm3)**
1	71	NSCLC,NOS #	III	RUL	62.1 cm3
2	64	NSCLC, Squamous Cell Cancer	III	LUL	119.5 cm3
3	49	Thymoma #	III		216.7 cm3
4	62	Small cell lung cancer	III	RUL	253.5 cm3
5	54	Thymoma #	III		260.7 cm3
6	52	Esophagus, Squamous Cell Cancer #	III		393.2 cm3
7	41	NSCLC, Squamous Cell Cancer	III	RLL	639.8 cm3
8	56	Esophagus, Squamous Cell Cancer	III		660.6 cm3
9	70	NSCLC, Squamous Cell Cancer	III	Carina bronchial	716.6 cm3
10	52	NSCLC, Esophagus, Squamous Cell Cancer #	III	LLL	732.8 cm3
11	72	NSCLC, Squamous Cell Cancer	III	LLL	804.9 cm3

The location is labeled as *RUL* = right upper lobe, *RLL* = right lower lobe, *LUL* = left upper lobe and *LLL* = left lower lobe. *NSCLC* = non-small cell lung cancer; *NOS* = not otherwise specified #: resection operation was performed before radiotherapy.

The 4D-CT data were obtained using the Varian real-time position management (RPM system, Varian Medical Systems, Inc. Palo Alto, CA) with respiratory motion tracked by an external marker. The collection and review of the clinical data for this project was approved by the ethical committee of the China Medical University Hospital, Taiwan (DMR100-IRB-216).

### IMRT planning

IMRT plans were generated on an Eclipse commercial treatment-planning system, version 8.1 (Varian Medical Systems) to deliver doses between 50–74 Gy to the PTV depending on diagnosis. The maximum intensity projection tool was used with images of all respiration phases to determine the internal gross target volume (IGTV). A margin of 5 to 10 mm was added to the IGTV to form the PTV. The time-averaged CT images [18] were used for normal structure delineation and dose calculation in planning. Two treatment plans were generated for each case. The first one, referred as anatomic plan, was generated without the incorporation of ventilation information. The goal was to provide a homogeneous dose to at least 95% of the PTV with maximal dose less than 110% of the prescribed dose and to restrict the relative volume of lung receiving a dose above 5 (V5), 10 (V10), and 20 Gy (V20) to be below 65%, 50%, and 35% respectively [19]. In the second plans, referred as functional plans in which ventilation information was incorporated, functional dose-volume constraints that account for the inhomogeneous function of lung were applied in IMRT planning. Regions of top 20% ventilation in the 4D-CT based ventilation image were set as avoid regions in the functional treatment plans. In addition, gantry angles in the functional plans were also changed from the anatomic plans to avoid the highly functional regions. Dose volume constraints for anatomic planning and functional planning are listed in Table 2. All the final anatomic and functional IMRT plans are clinically acceptable in this study.

**Table 2:** Dose volume constraints for anatomic planning and functional planning used for intensity-modulated radiotherapy (IMRT)

		**Anatomic planning**			**Functional planning**		
**Structure**	**Constraint type**	**Dose**	**Volume (%)**	**Priority**	**Dose**	**Volume (%)**	**Priority**
GTV	Maximum dose	105%	0%	50	105%	0%	50
	Minimum dose	103%	100%	450	103%	100%	450
CTV	Maximum dose	105%	0%	400	105%	0%	400
	Minimum dose	102%	100%	400	102%	100%	400
PTV	Maximum dose	105%	0%	650	105%	0%	650
	Minimum dose	101%	100%	350	101%	100%	350
Total lung	Maximum dose	5 Gy	30%	200	5 Gy	30%	200
	Maximum dose	10 Gy	25%	200	10 Gy	25%	200
	Maximum dose	20 Gy	20%	200	20 Gy	20%	200
Functional lung	Maximum dose				5 Gy	30%	250
	Maximum dose				10 Gy	25%	250
	Maximum dose				20 Gy	20%	250
Spinal cord	Maximum dose	40 Gy	0%	300	40 Gy	0%	300
Spinal cord + 5 mm	Maximum dose	45 Gy	0%	300	45 Gy	0%	300

### Deformable image registration

DIR provides a voxel-to-voxel deformation matrix among CT images from different phases of the respiratory cycle. The deformation matrix is used to quantify the density change within a particular voxel over the time course of the respiratory cycle. We previously developed the DIR method based on optical flow method [20] for tumor motion estimation across 4D-CT [21,22]. The validation was reported with 1 mm accuracy [22].

### Ventilation image

Pulmonary ventilation *P* can be defined as the fractional volume change in respiration [23,24]. It is expressed mathematically as

(1) P=ΔV-V

where *V* is the local volume at expiration and *ΔV* is the volume change from expiration to inspiration.

Considering two extreme phases of CT image sets among 4D-CT data, one taken at normal end expiration and the other at normal end inspiration, the volume of each voxel in expiration is a constant, determined by the CT voxel size. The voxel volume in expiration is expanded in inspiration. The boundary of a cube defined by 8 voxels in the expiration image set is deformed and no longer the same in the inspiration image set. DIR determines the new boundary location. The volume calculation program calculates the volume of the polyhedron deformed from the cube. The volume change *ΔV* is the volume difference between expiration and inspiration.

Paired CT images from 4D-CT at the normal end expiration and normal end inspiration phases were used for ventilation computation. In the current study, the definitions of highly-functional lung volumes were divided into top 20%, 30% and 40% in a ventilation image, respectively.

### Treatment plan analysis

The concepts of mean lung dose (MLD), V5 and V20 are no longer just applied to the total lung, but also applied to the high ventilation lung volumes of top 20%, 30% and 40% respectively. The 3D dose distributions from the anatomic IMRT plans were overlapped with the 3D ventilation distributions and analyzed. The analysis included the comparison of the MLD, V5 and V20 of the high ventilation volumes to that of the total lung. The differences between anatomic planning and functional planning on the dose and functional lung volume in small and large tumor groups were tested for significance using the Student’s *t*-test. Results were considered significant at *p* < 0.05.

Comparison of 90% conformity index (CI) and homogeneity index (HI) between anatomic and functional plans were analyzed. The CI, a measure of dose conformity, is defined as

(2) $$\mathrm{CI}=\frac{\mathrm{PT}V_{\mathrm{PI}}^{2}}{V_{\mathrm{PI}}\times \mathrm{PTV}}$$

where *PI* means the prescription isodose which was 90% of the prescription dose in this study, *PTV*_*PI*_ is the volume in PTV that is covered by *PI*, *V*_*PI*_ is the total volume, including PTV and normal tissue, that is covered by *PI*. The HI, a measure of dose homogeneity, is defined as

(3) $$\mathrm{HI}=\frac{D_{2\%}-D_{98\%}}{D_{P}}$$

where *D*_2%_ is the high dose that covers 2% of PTV volume, *D*_98%_ is the dose that covers the rest 98% of PTV and *D*_*P*_ is the prescription dose.

## Results

**Figure 1:**
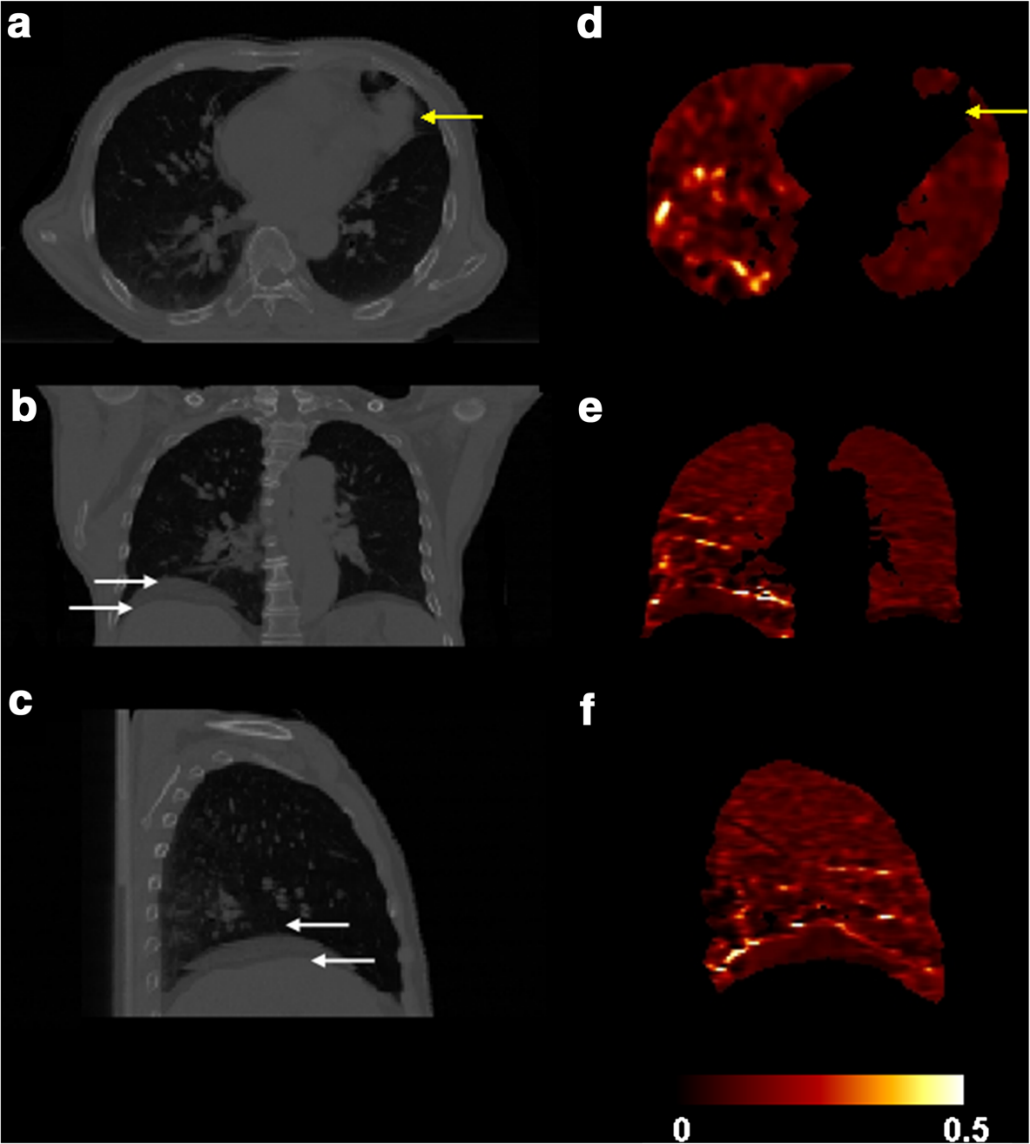
**The transverse (a), coronal (b) and sagittal (c) views of a 4D-CT.** The arrow indicated the location of the tumor. Corresponding views of 50%-0% (expiration-inspiration) ventilation of this example are shown as in (**d**), (**e**) and (**f**). Low ventilation is noticeable in the tumor region (indicated by the arrow on the CT image) on the ventilation image. The arrows on the CT image set indicate that the right diaphragm motion range was large. This volume change difference can be observed on the ventilation image. The ventilation image is scaled as *ΔV/V* = 0 as black and 0.5 as white.

Figure 1 shows the difference between two extreme phases, normal end inspiration phase and the normal end expiration phase for a 4D-CT image set of a lung cancer patient in transverse (a) coronal (b) and sagittal (c) views. The motion of the diaphragm can be observed in Figure 1b and c (arrows) and the corresponding ventilation images are shown in Figure 1d ~ f. In this example, different volume changes between left and right lungs are indicated by the diaphragm motion range difference in the overlapping CT images of the end expiration and inspiration phases. The 3 cm motion of the right diaphragm and 0.5 cm motion of the left diaphragm were observed. The ventilation difference of the left and right lungs can be seen on the corresponding ventilation image. Lower ventilation around lung tumor region was also observed in this example (Figure 1d).

**Figure 2:**
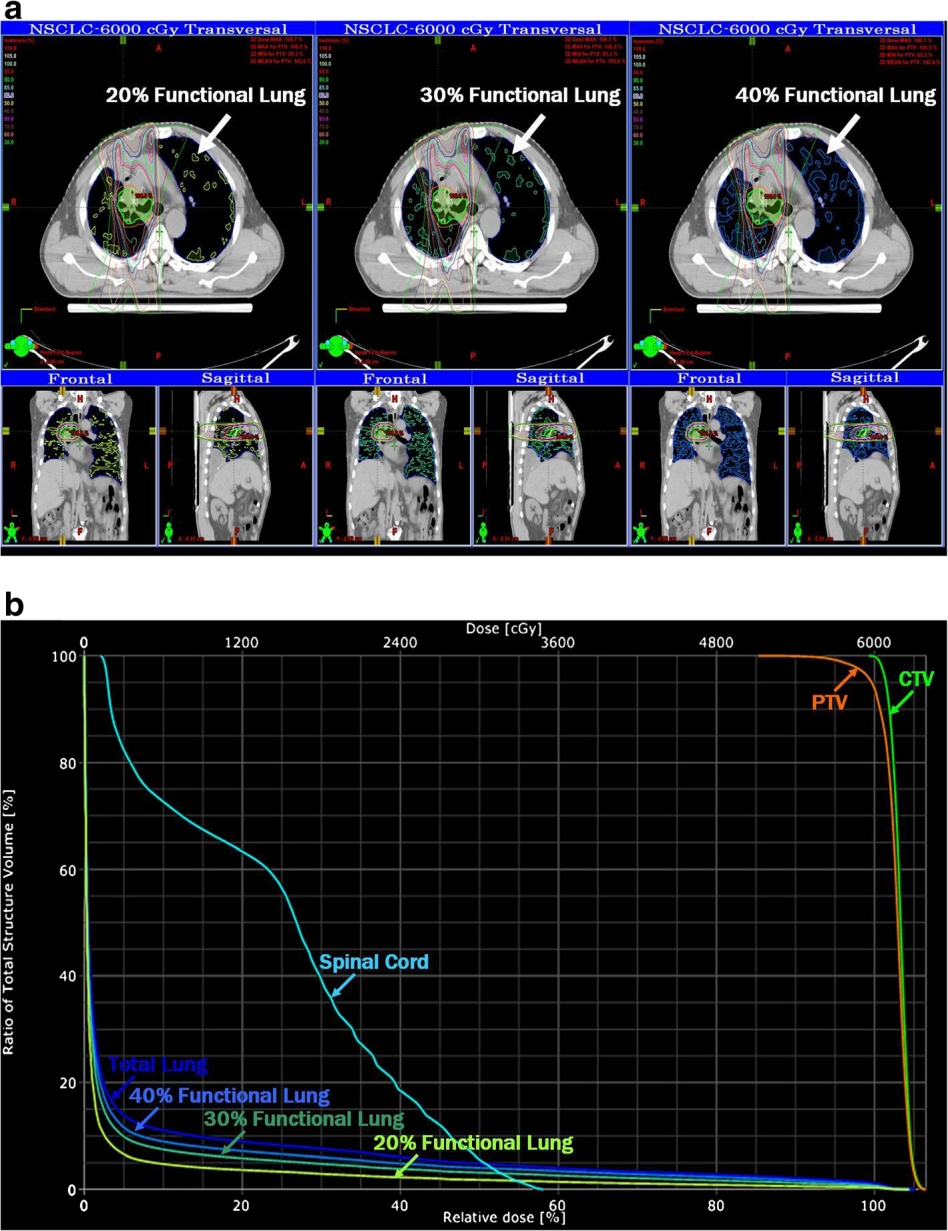
(a) Dose distribution of an anatomic IMRT treatment plan with high ventilation regions, as an example (b) The dose–(functional) volume histogram (DVH or DFVH) of the highly-functional lung region, at 20%, 30% and 40% for a large volume of NSCLS cancer patient.

Figure 2a shows the overlap of highly-functional lung volumes (ventilation of top 20%, 30% and 40%) on an anatomic IMRT plan as an example. The corresponding dose-volume histogram (DVH) was shown on Figure 2b. All anatomic IMRT plans show that a relatively homogeneous dose in the target volume wasachieved with the high dose regions conform to the target. The comparison between the large tumor and the small tumor subgroups shows that more critical structure sparing was achieved in the small tumor plans than in the large tumor plans.

**Figure 3:**
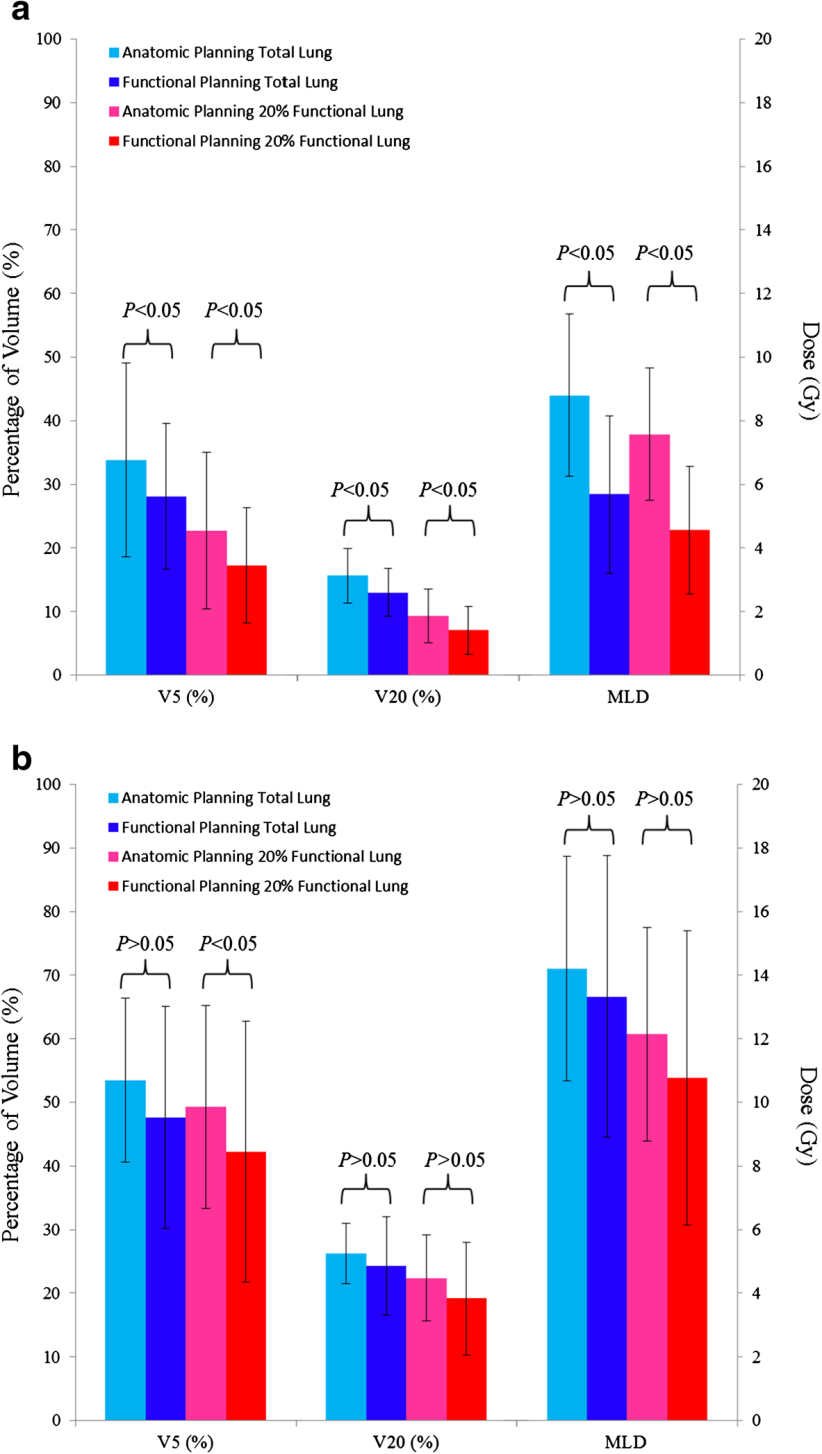
(a) Comparison of dosimetric parameters between anatomic and functional plans for small tumor cases and (b) for large tumor cases.

Table 3 lists the values of V5, V20 and MLD in total lung and top 20%, 30% and 40% functional lung regions for all the investigated anatomic treatment plans. The IMRT plan constraints, which included the radiation dose for lung V20≦30-35% and V5≦70% (NCCN Clinical Practice Guidelines in Oncology Non-Small Cell Lung Cancer version 2.2012), were all reached. For the cases of small target volumes (6 cases in total), all V5, V20 and MLD values for the highly-functional regions, top 20%, 30% and 40% ventilation, were lower than the values for the total lung. For the cases of large target volumes, two out of five cases had lower V5 and V20 values (Case 9, 11) in the highly-functional regions (top 20%) compared with the total lung. For the small tumor volume subgroup, when compared to the anatomic plans, the decrease in percentage of volume and dose for V5, V20 and MLD in total lung and the top 20% functional lung regions in the functional plans was statistically significant as demonstrated in Figure 3a. For the large tumor subgroup, the significant difference only appears on the percentage of volume for V5 in the top 20% functional lung regions (Figure 3b).

**Table 3:** Values of V5, V20 and mean lung dose (MLD) in total lung and top 20%, 30% and 40% functional lung regions for all the investigated anatomic treatment plans

**Patient**	**Anatomic**			**Functional lung region**								
	**Total lung**			**20%**			**30%**			**40%**		
	**V5 (%)**	**V20 (%)**	**MLD (Gy)**	**V5 (%)**	**V20 (%)**	**MLD (Gy)**	**V5 (%)**	**V20 (%)**	**MLD (Gy)**	**V5 (%)**	**V20 (%)**	**MLD (Gy)**
1	11.6	7.1	3.8	5.1	2.6	1.6	7.9	4.4	2.6	9.4	5.6	3.2
2	26.74	16.9	9.3	17.9	9.7	5.5	21	11.8	6.7	22.9	13.4	7.6
3	50.6	18.6	11.07	40.6	15.8	9.4	42.6	17	9.9	42.6	17.4	10
4	25.2	17.8	9.4	16.1	10.6	5.8	19.6	13.3	7.2	22.3	15.5	8.3
5	47.3	16.8	10.3	29.2	8.3	6	36.2	12	7.9	40.1	13.9	8.8
6	41.9	16.2	8.6	27	8.5	5.4	33.9	11.9	6.8	38.1	14.2	7.7
AVG ± STD	33.9 ± 15.1	15.6 ± 4.2	8.7 ± 2.6	22.7 ± 12.3	9.3 ± 4.3	5.6 ± 2.5	26.9 ± 12.9	11.7 ± 4.1	6.9 ± 2.4	29.2 ± 13.1	13.3 ± 4.1	7.6 ± 2.3
7	66.4	30.3	16.7	67.4	31.2	17.1	67.1	30	16.8	67.1	30	16.7
8	34.4	18.3	8.7	38.3	21.8	9.9	36.7	20.4	9.3	36.4	20.2	9.3
9	57.7	25.7	13.2	45	19.3	10.2	51.5	23	11.9	55.5	25.1	12.9
10	62.1	27.2	14.4	64.1	26	14.1	62.3	25.3	13.7	61.1	25	13.6
11	46.7	29.5	17.8	31.4	13.3	9.2	37.4	18.5	12.1	40.9	22.1	14
AVG ± STD	53.5 ± 12.9	26.2 ± 4.8	14.2 ± 3.6	49.2 ± 15.9	22.3 ± 6.8	12.1 ± 3.4	51.0 ± 13.9	23.4 ± 4.5	12.7 ± 2.7	52.2 ± 13.1	24.5 ± 3.7	13.2 ± 2.5

The 90% CI and HI for each plan of all 11 cases are listed in Table 4. Table 5 presents the summary in comparisons of the doses of total lung, functional lung, heart, spinal cord and esophagus between the anatomic and functional plans from all cases. In terms of lung doses, functional plans had lower doses in average compared with those in anatomic plans. For other organs at risk (OAR), functional planning led to greater doses than anatomic planning. The low dose region in lungs (about 12 Gy) was clearly improved in the functional plans.

**Table 4:** The prescription dose, conformity index (CI), heterogeneity index (HI) and the number of monitor units (MU) in the anatomical and functional IMRT plans for the 11 patients

**Patient**	**Diagnosis**	**Prescription dose (cGy)**	**PTV (cm3)**	**Anatomic planning**				**Functional planning**			
				**PTV**				**PTV**			
				**90%CI**	**HI**	**MU**	**Mean (%)**	**90%CI**	**HI**	**MU**	**Mean (%)**
1	NSCLC	6000	62.1	0.34	0.08	741	102.6	0.31	0.11	999	102.6
2	NSCLC	7400	119.5	0.68	0.07	387	101.9	0.62	0.10	724	101.9
3	Thymoma	5400	216.7	0.56	0.10	513	102.3	0.53	0.15	605	102.3
4	SCLC	5400	253.5	0.56	0.14	551	102.2	0.47	0.13	1182	102.2
5	Thymoma	5040	260.7	0.57	0.12	517	102.4	0.52	0.15	705	102.4
6	Esophagus	5040	393.2	0.64	0.11	734	102.4	0.75	0.13	790	102.4
7	NSCLC	5000	639.8	0.66	0.14	1060	100.9	0.64	0.11	1163	100.9
8	Esophagus	5040	660.6	0.61	0.12	960	102.5	0.40	0.12	973	102.5
9	NSCLC	5000	716.6	0.67	0.09	867	101.9	0.60	0.13	1291	101.9
10	Esophagus + NSCLC	5040	732.8	0.67	0.09	962	102.0	0.66	0.12	1074	102.0
11	NSCLC	6000	804.9	0.61	0.10	1367	101.1	0.47	0.12	1302	101.1

$\mathrm{CI}=\;\frac{\mathrm{PT}V_{\mathrm{PI}}\mathrm{PT}V_{\mathrm{PI}}}{V_{\mathrm{PI}}\mathrm{PTV}}$

*CI* : Conformity index.

*PI* : prescription isodose line.

*PTV*_*PI*_ : planning target volume within the prescribed isodose volume PI;

*V*_*PI*_ : volume of the prescription isodose line;

*PTV* : planning targe volume.

$\mathrm{HI}\;=\;\frac{D_{2\%}\;-\;D_{98\%}}{D_{P}}$

*HI*: Homogenity index.

*D*_2%_: represent the doses to 2% of the PTV.

*D*_98%_: represent the doses to 98% of the PTV.

*D*_P_: Prescription Dose.

**Table 5:** Summary of lung dose and critical organs for anatomic and functional treatment plans among 11 thoracic cancer patients

**Metric**	**Anatomic planning**	**Functional planning**
Total lung		
V5 (%)	42.77 ± 16.97	36.97 ± 17.05
V20(%)	20.44 ± 6.99	18.12 ± 8.11
MLD (Gy)	11.24 ± 4.03	10.19 ± 4.36
20% Functional lung		
V5 (%)	34.76 ± 19.21	28.61 ± 19.45
V20(%)	15.22 ± 8.59	12.57 ± 8.85
MLD (Gy)	8.61 ± 4.35	7.38 ± 4.59
Heart		
V40 (%)	16.75 ± 15.05	21.87 ± 23.12
Mean dose (Gy)	16.7 ± 12.78	17.31 ± 13.95
Spinal cord		
Maximun (Gy)	41.58 ± 5.73	43.31 ± 4.5
Esophagus		
V35 (%)	20.36 ± 16.14	24.15 ± 17.36
V50 (%)	11.57 ± 16.95	11.57 ± 16.96
Mean dose (Gy)	15.34 ± 9.23	16.22 ± 9.31

Abbreviations:

*VX(%)*: volume of an OAR receiving ≧ X Gy; *MLD*: Mean lung dose; Data presented as mean ± standard deviation.

## Discussion

In this retrospective study, the ventilation image derived from 4D-CT was applied for evaluation of radiotherapy treatment plans in functional lung sparing. One way to compare functional lung sparing is to compare the MLD for total lung and highly-functional lung. Based on the cases studied, for small target volume cases, the MLD of total lung was always higher than that of highly-functional lung volumes (20% ~ 40%). The reason for this is that the volume around tumor is usually of low ventilation, and for small targets, radiation beams cover smaller normal lung volume, thus affect less highly-functional lung volume. However, the consistent results were not found in this study for large target volume cases. The number of cases that the MLD in highly-functional lung volumes was higher than that of the total lung was about the same as the number of cases that the MLD in highly-functional lung volumes was lower, since the beams to cover large target volumes would go through large normal lung volume, which inevitably include highly-functional lung volume. The difference between the MLD values in highly-functional lung volumes and in the total lung was not great. While Case 7 to 10 are typical examples, Case 11 is an exception. The big tumor in Case 11 was in the left lung. The radiation beams were arranged to avoid the right lung completely where most the high ventilation lung volume was in. Thus in this case, the MLD of total lung was obviously higher than that of the highly-functional lung volumes.

In treatment planning, clinicians always try to minimize the radiation dose to normal lungs to reduce toxicity, usually without functional lung information [25]. The dose in highly-functional lung regions determines the risk of complication and thus should be an important index of treatment planning quality. This retrospective study investigates the dose in highly-functional lung volumes in the treatment plans developed without incorporation of the functional lung information. For small tumors, V5, V20 and MLD of highly-functional lung regions were mostly lower than the values of total lung. This implies that even without incorporation of functional lung information in IMRT treatment planning, the dose in highly-functional lung regions is usually low. For large tumors, it varies case by case. But in general, radiation treatment affects functional lung more when the target volume is large. From this point of view, it is more critical to incorporate functional lung information in treatment planning for patients with large tumors. It may be more difficult to achieve certain goals in functional lung sparing for large target volumes due to anatomical limitations. When the target is small, functional lung sparing should be easier to accomplish because a more flexible beam arrangement is possible. This study has demonstrated this trend (Figure 3).

With the highly-functional lung sparing constraints, all cases showed better V5 in the functional plans (Figure 3). Most functional plans demonstrated significant improvement in V5, V20 and MLD in the top 20% high ventilation volumes. Based on the MLD to total lung, there is only one functional plan in which the MLD to total lung was higher in the functional plans by 0.05 Gy. The reason for this was that the tumor was oblong in shape and close to the esophagus. When the objectives of proper PTV coverage and highly-functional lung sparing in the functional plans were satisfied, the MLD to the total lung got slightly worse.

In general, all functional plans generated with incorporation of 4D-CT based ventilation image for all thoracic cancer patients were successfully within constraints (Table 2) and acceptable for clinical use. Among the 11 cases studied, when compared with the anatomic plans, both better CI and HI in the functional plan were found in one case, better CI and superior HI in the functional plan were found in 1 and 9 cases respectively. More cases in the anatomic plans had better CI (10 out of 11) while more in the functional plans had better HI (9 out of 11). However, the differences are within a small range with ΔCI < 0.15 and ΔHI < 0.03. The CI and HI differences showed no strong relationship with the tumor size but more related to the shape and location relative to critical organs. Since there are more constraints in the functional planning, one would expect worse CI and HI in functional plans when compared with the anatomic plans. This trend is not clear for HI in this study.

The dose to the other OARs, such as heart, spinal cord and esophagus, were relatively higher in the functional plans than that in the anatomic plans. Since the doses to the other OARs in the functional plans were within the planning constraints, they were not forced to be better than the anatomic plans. Tighter doses to the other OARs could be achieved if tighter dose constraints to other OARs were made. However, since there are more planning constraints in the functional planning, some compromise has to be made, which could be the doses to the other OARs. If tighter doses to the other OARs are not achievable, one needs to evaluate the risks of complications to the lung and other OARs and make decision which plan to use, the anatomic or functional plan.

A recent study showed that the poor initial ventilation close to the tumor may improve with treatment induced tumor shrinkage [26]. Although lower V20 and MLD are recommended to limit the risk of radiation pneumonitis in general [1], many other studies showed that the pulmonary injury and radiation dose had a weak correlation [17,27,28]. Hence, the hypothesis that sparing of the best functioning lung volumes is beneficial for the patients still needs to be verified by clinical data.

## Conclusion

This study has demonstrated the evaluation of radiation dose of IMRT treatment plans on functional lung volumes by incorporation of functional lung imaging from 4D-CT for thoracic cancer patients. Radiation treatments affect functional lung volumes more seriously on larger tumor cases than that of smaller ones. Thus it is more critical to arrange radiation beams for functional lung sparing for patients with large tumors. Anatomic and functional treatment plans with 4D-CT-based ventilation imaging for thoracic cancer patients including NSCLC, thymoma and esophaguys tumors are presented and were all clinical acceptable. With some compromise of dose to other critical organs, the dose reduction in highly-functional lung regions to reduce lung toxicity can be achieved.

## Competing interests

The authors declare that they have no competing interests.

## Authors’ contributions

TC: contributed the frame work of the project; participated data analysis; performed most data measurement and calculation; carried out programming; participated draft of manuscript. CY: participated data acquisition and provided patient contours. CR: provided clinical patient data and treatment prescriptions; guided treatment plans. JA: coordinated the collaboration. TC: participated data acquisition. GZ: refined manuscript. All authors read and approved the final manuscript.
